# Association Between Alcohol Use and HIV-Related Sexual Risk Behaviors Among Men Who Have Sex with Men (MSM): Findings from a Multi-Site Bio-Behavioral Survey in India

**DOI:** 10.1007/s10461-014-0699-x

**Published:** 2014-01-24

**Authors:** Diwakar Yadav, Venkatesan Chakrapani, Prabuddhagopal Goswami, Shreena Ramanathan, Lakshmi Ramakrishnan, Bitra George, Shrabanti Sen, Thilakavathi Subramanian, Harikumar Rachakulla, Ramesh S. Paranjape

**Affiliations:** 1 FHI 360, H-5 (Ground Floor), Green Park Extension, New Delhi, 110016 India; 2 Centre for Sexuality and Health Research and Policy (C-SHaRP), Chennai, India; 3 National Institute of Epidemiology, Chennai, India; 4 National Institute of Nutrition, Hyderabad, India; 5 National AIDS Research Institute, Pune, India

**Keywords:** Alcohol use, Sexual risk, Condom use, Men who have sex with men, MSM, HIV, India

## Abstract

This paper examines the association between alcohol use and HIV-related sexual risk behaviors among men who have sex with men (MSM). A cross-sectional bio-behavioral survey was conducted among 3,880 MSM, recruited using time-location cluster sampling from cruising sites in three Indian states. Nearly three-fifths of the participants reported alcohol use. Among frequent users (40 % of the sample), defined as those who consumed alcohol daily or at least once a week, 66 % were aged 25 years and above, 53 % self-identified as *kothi* (feminine/receptive), and 63 % consistently used condoms with male paying partners. Multivariate logistic regression demonstrated that frequent users were more likely to be aged 25 years and above, less likely to self-identify as kothi, and less likely to consistently use condoms with male paying (AOR = 0.7; 95 % CI 0.5–0.9) and male regular (AOR = 0.7; 95 % CI 0.6–0.9) partners. HIV prevention interventions for MSM need to provide tailored information on alcohol use-related sexual risk, especially for MSM in sex work and MSM with male regular partners.

## Introduction

Men who have sex with men (MSM) are categorized as a high-risk group for HIV by India’s National AIDS Control Organisation [1]. The national average HIV prevalence among MSM is 7.4 %, about 20 times higher than the average for the general population (0.31 %); one HIV sero surveillance site reported HIV prevalence among MSM as high as 15 % [2]. Such high HIV prevalence among MSM in India is primarily attributed to unprotected sex with multiple male and female partners [3]. Therefore, identifying and addressing risk factors that facilitate unprotected sex among MSM are vital to HIV prevention. Alcohol use is one such factor.

Alcohol use and its association with HIV-related sexual risk is well documented by studies in other countries [4–7], including studies on MSM [8–11]. Studies in India have found alcohol use to be associated with HIV-related sexual risk across a range of male populations—married men [12], male migrant workers [13], men from low income communities [14], male clients of female sex workers (FSWs) [15], and male patrons of wine shops [16]. However, little information is available on alcohol use among Indian MSM and its association with HIV-related sexual risk.

The available data shows relatively high prevalence of alcohol use among Indian MSM. The National Behavioral Sentinel Surveillance Survey among at-risk groups in India found alcohol use (at least once a week) to be relatively high among MSM (36 %), compared with FSWs (15 %) and male clients of FSWs (13 %) [17]. Similarly, cross-sectional studies across India have demonstrated high prevalence of alcohol use among MSM, ranging from 4 to 28 % [18–21].

Qualitative studies on Indian MSM have documented lack of condom use during anal sex under the influence of alcohol [22, 23]. At the time of writing this paper, only two quantitative studies [18, 24] on Indian MSM—one from Chennai and another from Mumbai—had explicitly reported findings associating alcohol use with sexual risk, but both did not find any significant association between alcohol use and sexual risk with male partners. The study on MSM in Chennai showed a significant association between weekly alcohol use to the point of being “buzzed/drunk” and unprotected sex with female partners, but not with male partners [18], while the Mumbai study could not find a statistically significant association between alcohol dependence and consistent condom use with male partners [24]. Also, these single-site studies had no information on biological proxy indicators of sexual risk—prevalence of HIV and sexually transmitted infections (STIs).

In order to address these gaps and contribute to the development of strategies that target alcohol-related sexual risk among MSM, we analyzed data from a bio-behavioral survey conducted among multi-site community-based samples of MSM. In this paper, we describe the prevalence and patterns of alcohol use among MSM and its association with HIV-related sexual risk behaviors, especially consistent condom use with male paying, regular and paid partners and female regular partners.

## Methods

### Data Source

Data for this analysis was drawn from the second round of a series of cross-sectional surveys, termed integrated behavioral and biological assessment, conducted in 2009–2010. The surveys were part of the evaluation of a comprehensive HIV prevention program—*Avahan*: the India AIDS Initiative, funded by the Bill and Melinda Gates Foundation and implemented in India’s four southern (Tamil Nadu, Andhra Pradesh, Karnataka and Maharashtra) and two north-eastern (Nagaland and Manipur) states. For this paper, we restricted the analysis to the three southern states of Andhra Pradesh, Tamil Nadu and Maharashtra. The overall design of the survey has been described in detail elsewhere [25, 26]. In order to obtain a representative sample (3,880 MSM) from cruising sites such as parks, public toilets, bus stands and local train stations, we used a two-stage time-location cluster sampling (TLCS) procedure. In general, TLCS is used to recruit hard-to-reach populations, including high-risk MSM [27, 28]; it has been used in other studies on Indian MSM [21, 29]. In the two-stage TLCS procedure adapted for the present study, primary sampling units (cruising sites) were selected in the first stage with probability-proportional-to-estimated-size of the MSM population visiting the cruising sites. Potential participants who were aged 18 years or older and had had any kind of sex (oral or anal) with other men in the past month were identified in the second stage through systematic random sampling to recruit eligible and willing persons.

Behavioral data were collected through an interviewer-administered questionnaire in three Indian languages (Tamil, Telugu and Marathi), and blood and urine samples were tested for HIV and STIs. Written informed consent was obtained from the participants. The Health Ministry Screening Committee of the Indian Council of Medical Research (ICMR) and local ethical committees of the implementing institutes of ICMR (National AIDS Research Institute, Pune; National Institute of Nutrition, Hyderabad; and National Institute of Epidemiology, Chennai), and the Protection of Human Subjects Committee of FHI 360 approved the study protocol.

### Measures

#### Alcohol Use

Participants were asked how often they had consumed alcohol in the past month, for which the response options were: every day/daily, at least once a week, less than once a week, not in the past month or never consumed. We categorized the MSM who reported consuming alcohol daily or at least once a week as ‘frequent’ users of alcohol. Those reporting alcohol consumption of less than once a week, not in the past month, or never consumed were categorized as ‘non-/infrequent’ users of alcohol. This categorization was based on the opinion of MSM community leaders, who believed that a man regularly drinking alcohol at least once a week can be labeled as a ‘frequent’ user of alcohol (since social drinking is less likely to take place every week in this context, where the sample predominantly comes from the lower socioeconomic group). Also, the previous studies on Indian MSM used a similar categorization—‘weekly alcohol users’ [18] and alcohol consumption ‘<once per week vs.> once per week’ [19]. While specific scales for alcohol consumption, such as the Alcohol Use Disorders Identification Test [30], are available, we used the relatively simple self-reported measure of frequency of alcohol drinking, since that information was collected as part of this study focusing on HIV-related risk among MSM. Notably, this paper provides new information on the prevalence and correlates of alcohol use and its association with sexual risk behavior among MSM.

#### Sociodemographic Characteristics

Participants were asked about their current age, literacy level, employment status, marital status and self-identity (such as, *kothi*, *double*-*decker*, *panthi* and *hijra*). Self-identities among MSM are usually based on gender expression and sexual role: kothis are usually feminine and receptive, panthis are masculine and insertive [31, 32], and double-deckers are both insertive and receptive [33]. Although hijras belong to a distinct sociocultural group and can be regarded as male-to-female transgender persons, they were included under the ‘MSM’ umbrella for this analysis because of their relatively small sample size in this study (2.5 % of the total sample).

#### Consistency in Condom Use with Different Types of Male and Female Partners

Participants were asked how often they used condoms during anal (or vaginal) sex with different types of male and female partners. For the purposes of the survey, the different types of partners were operationally defined as—(1) *regular partner:* the main partner to whom the participant feels committed to, such as spouse or lover; (2) *paying partner:* the person who paid the participant in cash or kind in exchange for sex; (3) *casual partner:* a stranger, friend or acquaintance with whom the participant had sex but did not consider that person as a regular or paying partner; (4) *paid partner:* the person to whom the participant paid in cash or kind for having sex.

#### Self-Risk Perception and Exposure to HIV Intervention

The survey covered information on self-risk perception of acquiring HIV infection and exposure to HIV intervention. A composite indicator of the participants’ exposure to HIV intervention was created: participants were classified as having been exposed to HIV intervention if in the previous year they had received any of the three core program services—(1) contacted by a peer educator/outreach worker, (2) received condoms from outreach workers, or (3) visited an STI clinic—from a nongovernmental organization working with MSM.

#### Current Membership in a Community-Based Organization

The participants were asked if they were members of a community-based organization (CBO), that is, an agency that is formed and managed by self-identified MSM.

#### Prevalence of STIs and HIV

Blood and urine samples were collected and tested for HIV and other STIs. Syphilis was screened by the rapid plasma reagin test and confirmed by treponema pallidum hemagglutination assay. Nucleic-acid amplification (Gen-Probe APTIMA Combo 2 by Gen-Probe Inc., San Diego, California) tests were conducted to screen for chlamydial and gonococcal infections. HIV-seropositivity was determined through a two-test algorithm using enzyme immunoassay (J. Mitra EIA Kit). Participants who tested positive for any one of the three STIs (syphilis, gonorrhoea and chlamydiasis) were categorized as ‘having any STIs’.

### Data Analysis

Bivariate analyses (Chi square tests) examined the relationship between alcohol consumption and sociodemographic and sexual risk behavior variables. Multivariate logistic regression was conducted to assess the degree of association between frequent alcohol consumption and sociodemographic characteristics and other contextual factors, including consistency in condom use with different types of male and female partners, exposure to any HIV prevention intervention, current membership in a CBO, and ‘having any STIs’ or HIV. Associations were considered significant for *p*-values less than 0.05 at 95 % confidence interval. Analyses were conducted in Stata version 12.0 [34].

For logistic regression analyses, some variables that initially had three or more categories were collapsed into meaningful binary categories to aid interpretation of results, especially if there were very low frequencies in certain categories of those variables. Hence, age group was categorized as ‘18–24 years’ and ‘25 years and above’; educational qualification as ‘illiterate’ and ‘literate’; and marital status as ‘never married’ and ‘ever married’. For occupational status, we categorized participants into two groups: ‘unemployed/student’ and ‘employed’, with the latter including self-employed, nonagricultural labor and government/private employees. Since kothis are generally more visible, well organized and have relatively better access to interventions than other MSM subgroups [35], we categorized the self-identities of MSM as ‘kothis’ and ‘non-kothis’, with the latter including panthis, double-deckers and bisexual-identified MSM.

We used exploratory logical model building in this study. The covariates included in multivariate logistic models were selected based on the available literature on correlates of alcohol and condom use (for example, HIV risk perception), and assumptions based on discussion with HIV program people (for example, exposure to HIV prevention intervention and current membership in an MSM CBO). Therefore, in general we used both ‘inclusion strategy’ (inclusion of alternative explanations for any associations) and ‘exclusion strategy’ (inclusion of control variables and elimination of spuriousness) [36].

## Results

### Sociodemographic Characteristics, Alcohol Use and Sexual Risk Behavior

Of the 3,880 MSM who were interviewed, nearly three-fifths of the participants were aged 25 years or above. The majority of MSM were literate (88 %), employed (89 %), never married (71 %) and self-identified as kothi (55 %). About two-thirds (69 %) reported having male paying partners and 57 % reported having current membership in an MSM CBO. Nearly three-fourths of the participants had been exposed to HIV prevention intervention. The proportions of MSM with ‘any STIs (excluding HIV)’ and HIV were 7 and 13 %, respectively (see Table 1).

**Table 1:** Proportion of MSM who are frequent and non-/infrequent users of alcohol by background, sexual risk and other characteristics (Integrated Behavioral and Biological Assessment Survey; 2009–10)

Characteristics	Percent	Alcohol use in the previous month		Test of association	
		Frequent users	Non-/infrequent users	Chi square	*p* value
Sample size	3,880	1,535	2,345		
Total %	100	39.6	60.4		
Age group (in years)				38.4	0.000
Mean (S.D.)	27.7 (7.5)	28.6 (7.6)	27.2 (7.4)		
18–24	39.4 (1,528)	33.4	43.3		
25–34	42.6 (1,655)	46.3	40.3		
35 and above	18.0 (697)	20.3	16.5		
Education				22.5	0.000
Illiterate	12.7 (495)	14.7	11.5		
Up to 8th grade	24.1 (936)	26.0	22.9		
9th–12th grade	49.8 (1,933)	48.3	50.8		
Any college education	13.3 (516)	11.0	14.8		
Occupation				56.9	0.000
Unemployed/student	10.8 (426)	7.6	13.1		
Self-employed	20.4 (794)	22.2	19.3		
Nonagricultural labor	25.2 (981)	26.2	24.6		
Government/private employee	24.9 (969)	22.7	26.4		
Others	18.3 (710)	21.1	16.4		
Marital status				12.1	0.002
Never married	70.6 (2,741)	67.7	72.6		
Currently married	28.3 (1,097)	30.9	26.6		
Widowed/divorced/others	1.1 (42)	1.4	0.8		
Self-identity				41.6	0.000
Kothi (feminine/receptive)	54.6 (2,120)	52.8	55.8		
Panthis (masculine/insertive)	13.3 (516)	11.9	14.2		
Double-deckers (insertive and receptive)	11.5 (446)	10.9	11.9		
Bisexuals	18.1 (7.3)	20.3	16.7		
Hijra	2.5 (95)	4.1	1.4		
Consistent condom use with female regular partner				5.4	0.020
Yes	14.5 (184)	11.7	16.4		
No	85.5 (1,087)	88.3	83.6		
Consistent condom use with male paying partner				29.7	0.000
Yes	69.6 (1,653)	63.3	73.8		
No	30.4 (722)	36.7	26.2		
Consistent condom use with male paid partner				1.1	0.290
Yes	76.8 (447)	74.8	78.5		
No	23.2 (135)	25.2	21.5		
Consistent condom use with male regular partner				29.9	0.000
Yes	68.1 (1,623)	61.5	72.2		
No	31.9 (761)	38.5	27.8		
HIV risk perception				3.9	0.046
No	75.1 (2,915)	73.4	76.3		
Yes	24.9 (965)	26.6	23.7		
Exposure to HIV prevention intervention				0.6	0.432
No	23.1 (895)	22.4	23.5		
Yes	76.9 (2,985)	77.6	76.5		
Current membership in a community-based organization				5.9	0.014
No	42.7 (1,656)	45.1	41.1		
Yes	57.3 (2,224)	54.9	58.9		
HIV status				0.1	0.762
HIV-negative	87.4 (3,392)	87.6	87.3		
HIV-positive	12.6 (488)	12.4	12.7		
Any STIs				0.6	0.423
No	93.2 (3,615)	92.8	93.4		
Yes	6.8 (265)	7.2	6.6		

*STI* sexually transmitted infection, *S.D.* standard deviation

In univariate analyses, nearly three-fifths (60 %) of the participants reported ‘any alcohol use’ (use of alcohol every day, at least once a week or less than once a week). About 40 % participants were frequent users, consuming alcohol once a day or at least once a week. The characteristics of frequent users and non-/infrequent users varied (see Table 1). Among frequent users, two-thirds (66 %) were aged 25 years and above, most (85 %) were literate, about a half (53 %) self-identified as kothis, and 78 % had been exposed to any HIV prevention intervention. Compared with non-/infrequent users, a smaller percentage of frequent alcohol users reported consistent condom use with male paying partners (74 vs. 63 %; *χ* ^2^ = 29.7; *p* < 0.01). A marginally lower proportion of frequent users reported having membership in an MSM CBO, compared with non-/infrequent users (55 vs. 59 %, *χ* ^2^ = 5.9; *p* < 0.05). The prevalence of HIV (12 vs. 13 %, *χ* ^2^ = 0.1; *p* = 0.76) and any one of the three STIs (7 vs. 7 %, *χ* ^2^ = 0.6, *p* = 0.4) was marginally lower (but not statistically significant) among frequent users than the non-/infrequent users.

### Multivariate Analyses: Predictors of Alcohol Use Among MSM

The results in Table 2 show that frequent users were more likely to be in the age group of 25 years and above (AOR = 1.3; 95 % CI = 1.1–1.6), employed (AOR = 1.5; 95 % CI = 1.2–1.9), ever married (AOR = 1.3; 95 % CI = 1.0–1.7), and exposed to any HIV prevention intervention (AOR = 1.2; 95 % CI = 0.9–1.4). However, they were less likely to use condoms consistently with male paying (AOR = 0.7; 95 % CI = 0.5–0.9) or regular (AOR = 0.7; 95 % CI = 0.6–0.9) partners, to be a current member of an MSM CBO (AOR = 0.8; 95 % CI = 0.7–0.9), and to have kothi identity (AOR = 0.7; 95 % CI = 0.6–0.8). There was no statistically significant association between frequent alcohol use and being HIV-positive or having any STIs (AOR = 0.9; 95 % CI = 0.7–1.1).

**Table 2:** Association between frequent alcohol use and sociodemographic and sexual risk behaviors among MSM (*n* = 3,880), adjusted for sociodemographic characteristics and exposure to HIV intervention

Variables	Frequent alcohol use in the previous month	
	UOR (95 % CI)	AOR (95 % CI)
Age group (in years)		
18–24	1.0	1.0
25 and above	1.5 (1.3–1.7)***	1.3 (1.1–1.6)***
Education		
Illiterate	1.0	1.0
Literate	0.7 (0.6–0.9)***	0.8 (0.6–0.9)**
Occupation		
Unemployed/student	1.0	1.0
Employed	1.8 (1.4–2.2)***	1.5 (1.2–1.9)***
Marital status		
Never married	1.0	1.0
Ever married	1.2 (1.0–1.4)***	1.3 (1.0–1.7)**
Self-identity		
Non-kothi-identified MSM	1.0	1.0
Kothi-identified MSM	0.8 (0.7–1.0)*	0.7 (0.6–0.8)***
Consistent condom use with male paying partner^a^		
No	1.0	1.0
Yes	0.6 (0.5–0.7)***	0.7 (0.5–0.9)***
Consistent condom use with male regular partner^b^		
No	1.0	1.0
Yes	0.6 (0.5–0.7)***	0.7 (0.6–0.9)***
Consistent condom use with male paid partner^c^		
No	1.0	1.0
Yes	0.8 (0.5–1.9)	1.1 (0.7–1.6)
Consistent condom use with female regular partner^d^		
No	1.0	1.0
Yes	0.6 (0.4–0.9)***	1.0 (0.7–1.5)
Exposure to HIV prevention intervention		
No	1.0	1.0
Yes	1.0 (0.9–1.2)	1.2 (0.9–1.4)**
Current membership in a community-based organization		
No	1.0	1.0
Yes	0.8 (0.7–0.9)***	0.8 (0.7–0.9)**
Either HIV or ‘any STIs’		
No	1.0	1.0
Yes	1.0 (0.8–1.2)	0.9 (0.7–1.1)

*UOR* unadjusted odds ratio, *AOR* adjusted odds ratio, *CI* confidence interval

* p < 0.10; ** *p* < 0.05; *** *p* < 0.001

^a^Analysis restricted to those who had male paying partners

^b^Analysis restricted to those who had male regular partners

^c^Analysis restricted to those who had male paid partners

^d^Analysis restricted to those who had female regular partners

## Discussion

We found a high prevalence of ‘any alcohol use’ and ‘frequent’ alcohol use among the MSM in this study, with frequent users reporting higher prevalence of inconsistent condom use with male paying and regular partners. Frequent users were more likely to be above 25 years of age, to have ever been married, and to have had exposure to HIV prevention intervention. Kothi-identified MSM and MSM with membership in a CBO were at decreased odds for frequent use of alcohol.

The prevalence of ‘any alcohol use’ among the MSM in this sample was nearly thirteen times higher than the national prevalence of ‘regular or any alcohol use’ in the general population, which ranges 4–7 % [37–39]. Our finding that nearly two-fifths of the MSM were ‘frequent users’ of alcohol was similar to the observations in other studies among Indian MSM, which had reported ‘weekly alcohol use to the point of being buzzed/intoxicated’ to be 28 % [18] and ‘alcohol dependence/abuse’ to be 16.7 % [24].

The other studies, conducted in Chennai and Mumbai, reported higher prevalence of unprotected anal sex during the last sexual encounter with a male partner among MSM who had ‘alcohol dependence’ [24] and unprotected vaginal sex in the previous 3 months among MSM who were ‘weekly alcohol users’ [18], respectively. Our findings are consistent with these two studies [18, 24]. In addition, we found that those who use condoms consistently with male paying and regular partners were less likely to be frequent users of alcohol; such specific information on the male partner type and the association between frequent alcohol use and consistent condom use was previously unavailable.

As we did not collect sexual event-level data on alcohol and condom use in this study, we cannot categorically state that alcohol influenced condom use with male paying and regular partners of frequent alcohol users. Other studies from developed countries have documented that MSM in sex work are often frequent users of alcohol, which increases their chances of being under the influence of alcohol during sex work, and hence the possible influence of alcohol on inconsistent condom use with male paying partners [40–43]. Regarding inconsistent condom use with male regular partners as well, the causal association cannot be proved. Event-level data on the temporal relationship between alcohol and condom use with different types of male and female partners could provide the necessary evidence for or against the influence of alcohol on condom use.

Our finding that MSM aged 25 years and above were more likely to consume alcohol frequently is consistent with other studies from India [18] and developed countries [44, 45]. HIV prevention interventions in India mostly seem to reach the younger MSM population [35]. Having better access to health-related information in general and support of peers could partly explain the relatively lower proportion of frequent alcohol users in the younger age group. Reportedly, the relatively older MSM are largely out of the reach of both MSM CBOs and other MSM in general [35]. In the present study, it is unclear why the frequent users of alcohol are more likely to be aged above 25 years. One possible reason could be that as MSM become older, the number of contacts with their peers may decrease and they may not subsequently receive adequate social and psychological support.

Being married to a woman was another independent factor that our study found to be significantly associated with frequent alcohol use; this is consistent with the findings of the study on MSM in Chennai [19]. A qualitative study has documented that once married, many MSM, who previously accessed services from MSM CBOs, decrease their interactions with CBO staff or stop coming to CBOs altogether to avoid discrimination from their peers for marrying a woman [46]. This decreased frequency of contact with HIV intervention/CBO staff and the possible mental health issues related to heterosexual marriage could be linked to frequent alcohol use among married MSM. These findings emphasize the need for providing outreach and HIV-related and psychological support services to married MSM, which could help reduce frequent alcohol use in this population.

We also found that membership in MSM CBOs protected the participants from frequent alcohol use. CBO membership seems to provide the MSM an opportunity to interact with other MSM and receive psychological and emotional support from peers. However, limited information is available on the effects of community membership or collectivization on mental health, alcohol use or consistent condom use among MSM in India [47]. More studies are needed to explore and understand whether and how community-based collectives empower MSM communities, contribute to HIV prevention and protect against risky behaviors such as frequent alcohol use and unprotected sex.

MSM in India experience multiple and complex challenges, including stigmatization and discrimination, which may put them at risk for alcohol use and unprotected sex [23, 48]. Even though identifying the various factors that could contribute to an individual’s alcohol consumption pattern may be beyond the scope of HIV prevention intervention projects supported by the government, screening for frequent/problematic alcohol use can be considered in the context of alcohol-related HIV risk.

Our study found that kothi-identified MSM were less likely to frequently use alcohol. HIV interventions in India mainly reach kothi-identified MSM as they are relatively more visible and, in general, willing to visit and use services of CBOs [23, 49]. Compared with other subgroups, kothis are more likely to have a well-connected network with their peers and receive relatively better social and psychological support from one another. In fact, a study from Chennai reported that panthi-identified MSM, who in general are not connected to the social support systems of MSM, were more likely to be ‘weekly alcohol users’ than kothis [18].

We did not find any statistically significant association between frequent alcohol use and being HIV-positive or having any STIs. One possible reason for this could be that the MSM at study sites, irrespective of their alcohol consumption patterns, had been receiving regular medical check-ups and syndromic management of STIs for nearly 5 years prior to the study [50].

The present study has certain limitations. First, even though we used TLCS, a probability-based sampling technique, to recruit MSM for the study, caution should be exercised in generalizing this study’s findings to other MSM populations in India; these findings can primarily be generalized to those MSM who visit cruising sites [51]. Second, like other studies on MSM [18, 24] in India, we did not have information on alcohol use before or during sexual activity; this information would have been useful in providing further evidence on the influence of alcohol on condom use. Third, use of single-item measure of frequency of alcohol use has been shown to be insufficient when compared with AUDIT tool [52]. Also, the 1-month timeframe for this item might have further weakened its psychometric property. Fourth, there was lack of a specific timeframe for measuring consistent condom use with different types of male and female partners, while the frequency of alcohol use had a timeframe (previous one month). Fifth, social desirability bias may have led some participants to underreport their alcohol use and sexual risk behaviors. Despite this possible social desirability bias, we found a statistically significant association between frequent alcohol use and sexual risk in this survey. Despite these limitations, this paper provides program-relevant information on alcohol-related HIV risk behavior among MSM who visit cruising sites. Being a multi-site study and having a larger sample size means it can be better generalized, which is a key strength of this study.

## Conclusion

Our findings have shown that frequent alcohol use is significantly associated with HIV-related risk behaviors among Indian MSM, especially with their male paying and regular partners. The study indicates the need for HIV interventions to screen MSM for alcohol use and establish referral services with alcohol dependence treatment and counseling programs. MSM should be educated about alcohol use-related sexual risk through one-to-one interactions and educational materials. In addition, connecting self-identified MSM with CBOs may help them in accessing the much-needed psychological and social support from other MSM, the lack of which could be a risk factor for alcohol use. Future studies need to use standardized instruments to capture comprehensive information on alcohol consumption (frequency, quantity, ethanol content, intoxication level and bingeing). Also needed are studies to understand the reasons behind frequent/problematic alcohol use among MSM and to examine whether and how these reasons are different from those among the general population, given the stigma associated with same-sex sexual behavior. Event-level data on alcohol and condom use with different types of male and female partners is also needed to design tailored interventions that address alcohol use-related sexual risk among diverse subgroups of MSM.
